# Supplementary material for: Assessment of knowledge, attitude, and practice of disposing and storing unused and expired medicines among the communities of Kathmandu, Nepal
Source: PLoS One. 2022 Aug 4;17(8):e0272635. doi: 10.1371/journal.pone.0272635 (PMC9352092; doi:10.1371/journal.pone.0272635)
Supplement: Attachment — Submitted filename: Comments to disposal.docx [file pone.0272635.s001.docx]

General comments

- The write of the manuscript has grammatical, spelling and punctuation errors to be corrected.

Specific comments

1. Title need to be modified as ‘Assessment of knowledge, attitude and practice of disposing and storing of unused and expired medicines among the communities of Nepal’.
2. Method

- Clustering is applied for multistage sampling techniques of large population. Why multi-staging was applied for your research? How many households are in each cluster?
- The quota sampling technique is not appropriate for selecting the households as there will be different size households in each cluster. It would have been good to use probability method of selecting the households in each cluster as the method of sampling and then sample size could be increased to increase representativeness of the data.
- The sample size determination would have been considering the design effect of multistage design to increase sample size. Why you did not consider it? Why did you determine to take 10 households from each cluster? It would have been good to include more households in the study.
- Make it clear whether the pilot study was conducted on the households under the cluster of interest.
- It would have been great to collect practice data, at least better to be supported, by observation method of data collection in each household.

3. Results

- The description of Table 1 is not in agreement with the one presented in the table (205 versus 19% of respondents in Bhaktapur cluster)
- After finishing the description of the findings, you have to put the referring table in the bracket as (Table …)
- The overall description of the findings of practice of disposing and storing medicines has redundancy. So, put it in summarized form

4. Discussion

- The introduction part of the discussion is not related to your findings rather focus on health insurance and about WHO guidelines. Therefore, please edit and discuss based on the findings you got in a clear and brief manner by comparing your findings with other similar literatures with justification.
- Based on the statistical analysis of knowledge and attitude, which group of people will be the target group for intervention strategies and discuss them very well.
