# Supplementary material for: Assessment of knowledge, attitude, and practice of disposing and storing unused and expired medicines among the communities of Kathmandu, Nepal
Source: PLoS One. 2022 Aug 4;17(8):e0272635. doi: 10.1371/journal.pone.0272635 (PMC9352092; doi:10.1371/journal.pone.0272635)
Supplement: Attachment — Submitted filename: Response to Reviewers 22nd July.docx [file pone.0272635.s003.docx]

**Revision letter**

Lalitpur, Nepal

Date: 21^st^ July 2022

To

The Editor-in-Chief

PLOS ONE

Sub: Submission of the revised version of the manuscript for consideration of publication

Dear Editor-in-Chief,

We are resubmitting the manuscript “Assessment of knowledge, attitude, and practice of disposing and storing unused and expired medicines among the communities of Nepal.” after revising it and making necessary corrections for consideration for publication in your esteemed journal. We are grateful to the reviewers for their constructive comments.

The response to specific comments is as follows:

Abstract

Comment 1- In page 55 of the manuscript file, on page 3 line 39 to 41, ‘The study explored the status of medicine disposal and storage practices and evaluated knowledge, attitude, and practice of medicine storage and disposal techniques among households of the three districts of the Kathmandu valley, Nepal.’ Please revise the sentence, as it stands it seems a bit confusing and contains repetitions.

Response: This has been corrected as:

The study evaluated knowledge, and attitude regarding unused and expired medicines and explored medicine storage and disposal practices among selected households in the Kathmandu valley, Nepal.

Comment 2- In page 55 of the manuscript file, on page 3, the sentences in Results subsection ‘The safe methods of medicine disposal were not known by 137 (65.2%) participants. Throwing in a dustbin was the preferred method of expired medicine disposal.’ Seem to be contradictory to the conclusion subsection ‘There is good level of knowledge and practice 58 of disposing the unused medicines among the public of Kathmandu Valley’ please explain why or correct the conclusion

Response: The statement has been corrected in the conclusion part as stated below.

The level of knowledge and practice of disposing of unused and expired medicines requires improvement.

Introduction

Comment 3 - At the end of the introduction section the objective stated ‘This study evaluates medicine disposal and storage practices and knowledge, attitude, and practice of medicine storage and disposal techniques among households residing in the three districts of the Kathmandu valley.’ Please make the objective presented at the end of the introduction section the same as the one in the abstract (of course after revising the sentence to be concise and clear)

Response: This has been changed as suggested.

The study evaluated knowledge, and attitude regarding unused and expired medicines and explored medicine storage and disposal practices among selected households in the Kathmandu valley, Nepal.

Methodology

Comment 4- ‘study area and period’: in this subsection the authors could consider providing a short introduction to Kathmandu to readers (as the readership of the journal is international) who may not be familiar with the area. For example, the authors could describe the general socio-demographic and health information of the area.

Response: A description of Kathmandu has been added as suggested at the beginning of the Methodology section.

Comment 5 - Was there a cut-off score to identify a respondent considered knowledgeable on medicine storage and disposal?

Response: We did not use any cut-off score to classify the knowledge score.

Comment 6- Have the authors considered conducting a regression analysis (for the knowledge and attitude scores) to assess the factors associated to them in a manner that will take account of potential confounders? This is crucial in determining which factors are really responsible for/predict knowledge or attitude after controlling for other potential confounders.

Response: Thank you. Since the knowledge score was only significantly different according to one of the socio-economic variables on the t-test/1-way ANOVA and other variables had a p-value > 0.25, it did not make sense to proceed with the multivariate analysis to find an independent effect controlling for confounders using regression analysis. Attitude score was found to be skewed and so an independent samples median test was used to compare it across socio-demographic variables, linear regression was not applicable for this data and thus it was not considered for the multivariate analysis.

**Results**

Comment 7- Please use subheadings to identify sections such as ‘Demographic characteristics’, ‘Knowledge’, ‘Attitude’ and ‘Practice’. This would help o low o ideas and for ease of following by readers.

Response: This has been added as suggested.

Discussion

Comment 8- I believe the discussion section still requires more work in terms of structure and flow of ideas. I find it difficult to follow the thread of thought in this section. In my opinion, starting by providing a summary of the main findings in the first paragraph and in the following paragraphs expounding on these findings by comparing with other studies and discussing implications might improve this section. In terms of order the themes and findings within knowledge, attitude and practice components could be presented in the sequential paragraphs.

Response: This has been corrected as suggested.

Conclusion

Comment 9- I notice a similar issue in the Conclusion where practice issues are presented before addressing knowledge.

Response: This has been addressed as suggested.

Comment 10- Please address this and be consistent in terms of ideas (i.e. I believe the findings should inform the discussion and conclusions).

Response: Thank you. This has been corrected as suggested.

Reviewer #3: Most of the suggested revisions were not carried out by the authors.

Examples:

Comment 11- Lines 301 -304 : Using medical and nursing students for comparison here may not be too appropriate because of their knowledge base about medicines versus the general public

Response: Thank you for the comment. The newly added references are for comparing the results with the general public and not with the nursing and medical students.

Comment 12- Lines 335-337: This statement can be re-phrased for better clarity

Response: This has been rephrased as: Another study mentioned that only 13.3% of respondents were aware of the proper medicine disposal methods and only 1% of participants knew about returning the medicines to the pharmacies for the medicine take-back systems.^25^

Comment 13- Lines 217-223: There is no need to repeat the information in the table in-text. One may highlight one or two important ones and then refer to the table.

Response: The corrected part is written as: The knowledge scores were significantly different only among respondents with different educational levels (p-value < 0.05). So, a pairwise comparison was done for this variable using Tukey’s HSD post-hoc test, and a statistically significant difference was observed between respondents with Secondary and University levels only (Table 3).

Comment 14- The tables to conform to the journal's specifications

Response: The tables are as per the guidelines of the journal.

Comment 15- Overall, the standard of English language needs to be improved upon

Response: The manuscript has been copyedited for language issues.

7. PLOS authors have the option to publish the peer review history of their article ([what does this mean?](https://journals.plos.org/plosone/s/editorial-and-peer-review-process#loc-peer-review-history)). If published, this will include your full peer review and any attached files.


If you choose “no”, your identity will remain anonymous but your review may still be made public.


**Do you want your identity to be public for this peer review?** For information about this choice, including consent withdrawal, please see our [Privacy Policy](https://www.plos.org/privacy-policy).

Reviewer #1: No

Reviewer #3: **Yes:** Prof. Joseph O. Fadare

Hoping for a favorable consideration

Thanking you

Yours Sincerely,

Nisha Jha and coauthors

Professor, Department of Pharmacology

KIST Medical College

Imadol, Lalitpur, Nepal

E-mail: nishajha32@gmail.com

Phone: 00977-01-5201680

Fax: 00977-01-5201496
